# Clinical application of voriconazole in pediatric patients: a systematic review

**DOI:** 10.1186/s13052-024-01684-z

**Published:** 2024-06-09

**Authors:** Lin Hu, Juanjuan Huang, Yanfei Li, Gefei He

**Affiliations:** 1 Department of Pharmacy, The Affiliated Changsha Hospital of Xiangya School of Medicine, Central South University, Changsha, Hunan China; 2 Department of Pharmacy, The First Hospital of Changsha, Changsha, Hunan China

**Keywords:** Voriconazole, Pediatric patients, Plasma trough concentrations, Factors, Optimal dose

## Abstract

The purpose of this study was to review the literature on the clinical use of voriconazole (VRC) in pediatric patients. MEDLINE, Embase, PubMed, Web of Science, and Cochrane Library were searched from January 1, 2000, to August 15, 2023 for relevant clinical studies on VRC use in pediatric patients. Data were collected based on inclusion and exclusion criteria, and a systematic review was performed on recent research related to the use of VRC in pediatric patients. This systematic review included a total of 35 observational studies among which there were 16 studies investigating factors influencing VRC plasma trough concentrations (C_trough_) in pediatric patients, 14 studies exploring VRC maintenance doses required to achieve target range of C_trough_, and 11 studies focusing on population pharmacokinetic (PPK) research of VRC in pediatric patients. Our study found that the C_trough_ of VRC were influenced by both genetic and non-genetic factors. The optimal dosing of VRC was correlated with age in pediatric patients, and younger children usually required higher VRC doses to achieve target C_trough_ compared to older children. Establishing a PPK model for VRC can assist in achieving more precise individualized dosing in children.

## Introduction

Voriconazole (VRC) is a broad-spectrum triazole antifungal agent, primarily used for the treatment of progressive and potentially life-threatening fungal infections, as well as for the prevention of invasive fungal infections (IFIs) in high-risk patients undergoing allogeneic hematopoietic stem cell transplantation (allo-HSCT) [1, 2]. The Infectious Diseases Society of America (IDSA) and the European Conference on Infections in Leukaemia (ECIL) all recommended VRC as the preferred treatment for invasive aspergillosis (IA) [3, 4].

VRC is rapidly and completely absorbed through oral administration, and it is widely distributed in tissues. However, due to its narrow therapeutic window and significant inter- and intra-individual variability in plasma trough concentrations (C_trough_) [5], personalized dosing strategies should be implemented to ensure efficacy and reduce adverse reactions. In recent years, there have been numerous studies related to VRC therapeutic drug monitoring (TDM), population pharmacokinetics (PPK) analysis and pharmacogenomics in children. Research on the factors affecting VRC C_trough_ and dose optimization has been constantly being updated.

As a special population in terms of medication, ensuring the safety and efficacy of VRC use is of utmost importance in pediatric patients. The VRC use in pediatric patients has gained the increasing attention of researchers. There were currently many studies on VRC use in children. However, without summarizing these findings, clinicians or pharmacists may lack sufficient understanding of the characteristics of VRC use in pediatric patients, potentially hindering the achievement of personalized dosing.

Therefore, we need to summarize the research on pediatric VRC use. The aim of this review was to provide guidance for improving the effectiveness and safety of VRC in pediatric patients and to establish a theoretical basis for achieving personalized dosing in clinical therapeutics.

## Methods

The authors identified three key questions:

i. What dosage is required to attain the target C_trough_ of VRC?
ii. What factors influence VRC C_trough_ in pediatric patients?
iii. What recommendations can be derived from the PPK study of VRC for personalized medication?

### Search strategy

We conducted a systematic review in accordance with the guidelines outlined in the Preferred Reporting Items for Systematic Reviews and Meta-analyses (PRISMA) [6]. We conducted computer searches in databases, such as MEDLINE, EMbase, PubMed, Web of science and Cochrane Library databases, with a search period spanning from January 1, 2000, to August 15, 2023. Duplicate articles found in different databases were removed by Using EndNote. Based on the characteristics of different databases, corresponding search strategies were formulated to preliminarily screen literature related to the use of VRC in pediatric patients. The search terms were as follows: (voriconazole) AND (children) OR (child) OR (pediatric patient) OR (infant) OR (adolescent) AND (factor) OR (influence) OR (affect) OR (effect) OR (population pharmacokinetic) OR (PPK) OR (dose optimization) OR (dosage optimization).

### Study selection

All articles describing factors influencing VRC C_trough_, dose optimization and PPK studies were included in this review. The inclusion criteria: (1) the study drug must be VRC, and steady-state C_trough_ must be monitored. (2) the study population must contain patients aged 0 to 18 years. (3) articles must be written in English. The exclusion criteria: (1) in vitro and animal studies. (2) reviews, systematic reviews, meta-analyses, letters, comments or case reports.

### Data extraction

According to the purpose and specific content of this review, a uniform data extraction table was formulated. Two authors recorded the following information of included studies: authors, publication dates, countries, study design, sample sizes, patient characteristics such as underlying diseases and range of age, target range of VRC steady-state C_trough_, factors significantly influencing VRC C_trough_, VRC dosages, administration routes, durations of VRC use, software and models used in PPK studies, significant covariates affecting pharmacokinetic (PK) parameters and main results or conclusions of dose simulation experiments. Any disputed issues were discussed and resolved by the third author. We would not conduct further statistical analysis of the research data mentioned in this review and the results were displayed in tables.

### Quality evaluation of studies

Observational studies were evaluated for adherence to the Strengthening the Reporting of Observational Studies in Epidemiology (STROBE) guidelines [7].

## Results

### Study selection

**Fig. 1:**
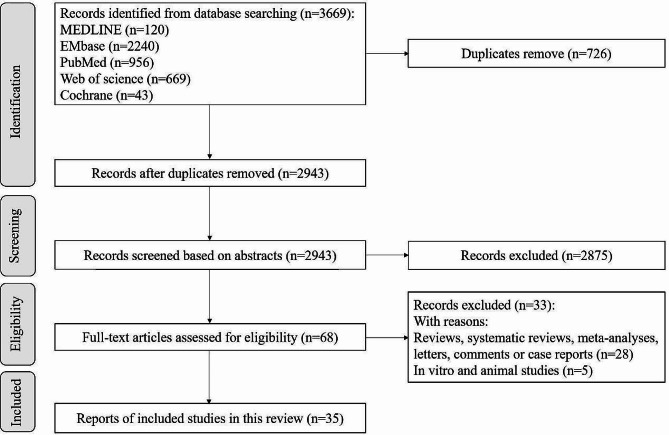
The flowchart of articles selection

**Fig. 2:**
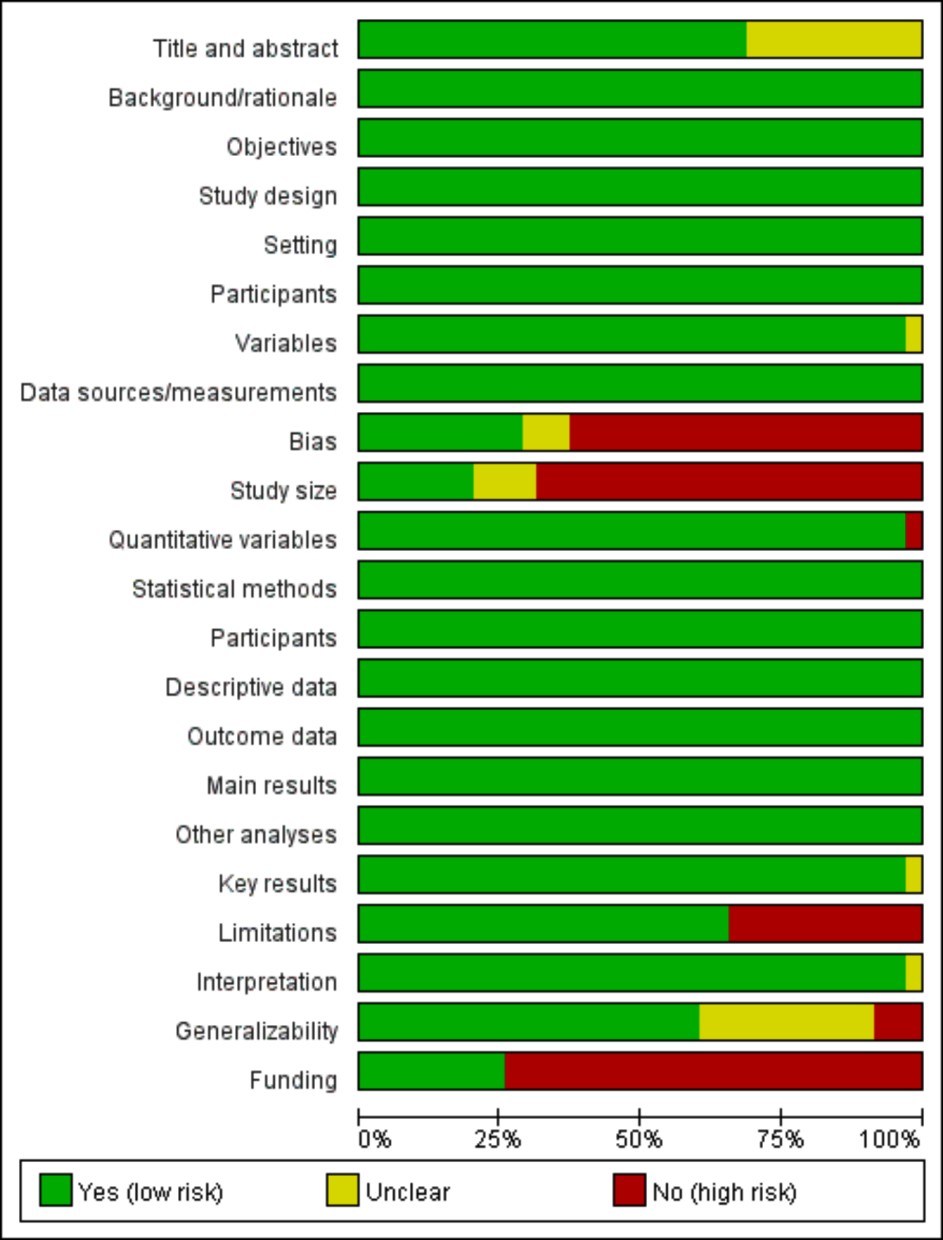
Adherence to STROBE recommendations

A total of 3 669 relevant articles were searched from the database (120 from MEDLINE, 2 240 from Embase, 956 from PubMed, 669 from Web of Science, and 43 from the Cochrane Library). According to the criteria of inclusion and exclusion, a total of 35 observational studies [8–42] remained in the systematic review after excluding 3 634 articles. The process and outcomes of literature screening were presented in Fig. 1, while the quality assessment of the selected studies was reported in Fig. 2.

### What dosage is required to attain the target C_trough_ of VRC?

The maintenance doses of VRC required to achieve target range of C_trough_ in both Asian (7 published studies) and non-Asian (7 published studies) pediatric patients were significantly correlated with age, as detailed in Tables 1 and 2. Boast et al. [20] found that due to the higher clearance rate (CL) and larger apparent volume of distribution in younger children compared to older children, the median intravenous dosages required to achieve target C_trough_ for Australia patients aged < 6, 6–12 and > 12 years were 8.8, 7.5 and 4.0 mg/kg twice daily, respectiveiy (*P* < 0.001). Bartelink et al. [18] discovered that the average dosages required to achieve target C_trough_ in Dutch children aged < 2, 2–12 and > 12 years were 31.5, 16.0 and 9.4 mg/kg/day, respectively, with statistically significant differences in daily dosages among the three age groups. Similar results were observed in our previous studies involving Chinese pediatric patients [11, 14]. The above researches all found that younger children required higher doses to achieve the target VRC C_trough_ compared to older children. Therefore, both Asian and non-Asian pediatric patients required individualized VRC dosing regimens based on age.

**Table 1:** Summary of studies on VRC maintenance doses to achieve the target range in Asian populations

Study population	No. of samples	Target C_trough_ (mg/L)	Maintenance dose to achieve the target range		Year	Country	Reference
			Age group (years)	Administration routes and VRC Dose (median [range], mg/kg twice daily)			
Pediatric cancer patients with IA	27	1.0–6.0	< 12 ≥ 12	PO 6.3 ^a^ IV 5.6 ^a^ PO 4.1 ^a^ IV 4.1 ^a^	2013	Korea	Choi et al. [8]
Pediatric patients with tumor	20	1.0–5.0	≤ 5 6–12 ≥ 13	PO 15.05 ^a^ IV 6.55 ^a^ PO 4.75 ^a^ IV 4.75 ^a^ PO 4.35 ^a^ IV 2.75 ^a^	2016	Japan	Kato et al. [9]
Children with immunodeficiencies	107	1.0-5.5	0–12	IV 5- < 7	2017	China	Liu et al. [10]
Children with hematological diseases	42	1.0-5.5	< 6 6–12 > 12	PO 11.1 (6.7–13.8) PO 7.2 (4.2–10.3) IV 5.8 (5.0-7.7) PO 5.3 (4.0-8.5) IV 4.9 (3.6–6.3)	2018	China	Hu et al. [11]
Children with hematological diseases	108	0.5-5.0	*CYP2C19* NMs and ≤ 12 *CYP2C19* NMs and > 12 *CYP2C19* IMs/PMs and ≤ 12 *CYP2C19* IMs/PMs and > 12	6.53 ± 2.08 ^b^ 3.95 ± 0.85 ^b^ 5.75 ± 1.73 ^b^ 4.23 ± 0.76 ^b^	2021	China	Tian et al. [12]
Immunocompromised children	91	1.0–5.0	*CYP2C19* NMs *CYP2C19* IMs *CYP2C19* PMs	10.4 (8.1–13.4) 9.1 (6.65–10.9) 7.6 (5.35–9.55)	2022	China	Chen et al. [13]
Children with hematological diseases	131	1.0-5.5	2–14	PO 4.75 ± 2.05 ^b^ IV 5.21 ± 1.81 ^b^	2023	China	Hu et al. [14]

VRC, voriconazole. C_trough_, trough concentration. PO, oral. IV, intravenous. NMs, normal metabolizers. IMs, intermediate metabolizers. PMs, poor metabolizers. IA, invasive aspergillosis. ^a^ median dose. ^b^$\bar x \pm s$

**Table 2:** Summary of studies on VRC maintenance doses to achieve the target range in non-Asian populations

Study population	No. of samples	Target C_trough_ (mg/L)	Maintenance dose to achieve the target range		Year	Country	Reference
			Age group (years)	Administration routes and VRC Dose (median [range], mg/kg twice daily)			
Infants and children with primary immunodeficiency	16	> 1.0	0–14	10–16	2011	France	Gerin et al. [15]
Immunocompromised children	30	1.0-5.5	< 5 ≥ 5	19 (6–20) 7.5 (2–26)	2012	Spain	Soler-Palacín et al. [16]
Immunocompromised paediatric patients	74	2.0–5.0	0.2–18	6.45 ± 2.85 ^b^	2012	Germany	Pieper et al. [17]
Children with HSCT	61	1.0–5.0	< 2 2–12 > 12	IV 15.75 (6-35.5) PO 11 (7–15) IV 7.75 (6.5–27.5) PO 4.3 (4-7.5) IV 5.95 (4.5–10)	2013	Netherlands	Bartelink et al. [18]
Children with IFIs	11	1.0–6.0	2–12	5–7	2015	America	Tucker et al. [19]
Immunocompromised children	55	1.0–5.0	< 6 6–12 > 12	IV 8.8 ^a^ PO 4.7 ^a^ IV 7.5 ^a^ PO 4.3 ^a^ IV 4.0 ^a^	2016	Australia	Boast et al. [20]
Children received VRC for at least 48 h	59	1.0–6.0	< 12 ≥ 12	11.15 (9.00-13.55) ^c^ 6.0 (4.9-7.0) ^c^	2023	America	Zembles et al. [21]

VRC, voriconazole. C_trough_, trough concentration. PO, oral. IV, intravenous. HSCT, hematopoietic stem cell transplantation. IFIs, invasive fungal infections. ^a^ median dose. ^b^$\bar x \pm s$, ^c^ median [interquartile range]

We have also observed that the maintenance doses of VRC required to achieve target C_trough_ might differ between Asian and non-Asian pediatric patients. A retrospective study from China found that intravenous dosages of 5–7 mg/kg twice daily could satisfy the requirements for achieving target C_trough_ in most Asian pediatric patients [10]. Hu et al. [11] also discovered that the oral and intravenous dosages needed to achieve target C_trough_ in pediatric patients were significantly lower than the recommended dosages in European or American package inserts (7.7 mg/kg vs. 9 mg/kg, *P* = 0.033; 5.6 mg/kg vs. 8 mg/kg, *P* = 0.003). However, due to the unavailability of data for further statistical analysis, it remains uncertain whether differences exist in the VRC doses needed to achieve target C_trough_ between Asian and non-Asian pediatric populations.

### What factors influence VRC C_trough_ in pediatric patients?

The C_trough_ of VRC were influenced by various factors in pediatric patients. Currently, there have been 16 published studies investigating the determinants of VRC C_trough_ in pediatric patients, among which only 2 were prospective studies, while the rest were retrospective, single-center and descriptive studies. Those studies came from various regions: Asia (*n* = 11; 68.8%), encompassing 9 from China, 1 from Japan, and 1 from Korea; Europe (*n* = 4; 25.0%), comprising two in Italy and one each in Spain and Switzerland; and South America (*n* = 1; 6.2%), specifically from Chile. Six studies had included sample sizes of over 100 pediatric patients, and merely two studies had encompassed sample sizes exceeding 200 pediatric patients. These investigations have identified more than ten factors that could significantly impact VRC C_trough_, as outlined in Table 3. The most frequently reported significant influencing factors including CYP2C19 genetic polymorphism, co-administration of proton pump inhibitors (PPIs), inflammation status and liver function indicators.

**Table 3:** Summary of studies exploring the factors affecting the VRC C_trough_ in pediatric patients

Study design	Age (years)	No. of samples	Year	Country	Main results and conclusions	Reference
Retrospective, single-center study	0–18	20	2016	Japan	Younger age and oral administration were significantly associated with lower VRC C_trough_.	Kato et al. [9]
Retrospective, single-center study	0–12	107	2017	China	The co-administration of omeprazole significantly increased VRC C_trough_. There was a significant positive correlation between VRC C_trough_ and Scr levels, and a negative correlation with ALB levels.	Liu et al. [10]
Retrospective, single-center study	< 18	237	2018	Italy	There was a positive correlation between VRC dose and plasma exposure. Patients with higher Scr levels had higher VRC C_trough_. Additionally, there was a positive correlation between VRC C_trough_ and age. Males exhibited higher median C_trough_ than females.	Allegra et al. [22]
Retrospective, single-center study	< 18	232	2018	Italy	SLCO1B3 rs4149117 c.334 GT/TT, ABCG2 rs13120400 c.1194 + 928 CC and ABCC2 rs717620 c.-24 GA/AA genotype significantly affected VRC C_trough_.	Allegra et al. [23]
Retrospective, single-center study	2–14	42	2018	China	Intravenous administration and co-administration of PPI significantly increased initial VRC C_trough_.	Hu et al. [11]
Retrospective, single-center study	< 18	33	2019	Chile	Patients with carriers of the CYP2C19*17 polymorphism (rs12248560) variant presented significantly lower VRC C_trough_ than non-carriers.	Espinoza et al. [24]
Retrospective, single-center and cohort study	< 18	61	2020	Korea	Oral administration and CRP levels were associated with low initial VRC C_trough_. ALT levels were associated with a high initial VRC C_trough_.	Kang et al. [25]
Non-interventional retrospective clinical study	2–18	94	2021	China	Age, WT, dose, DBil, BUN and CYP2C19 phenotypes were found to be influencing factors of VRC C_trough_.	Zhao et al. [26]
Retrospective, single-center study	< 18	108	2021	China	Age, combination medication with PPIs and CYP2C19 phenotype accounted for some of variability in VRC C_trough_.	Tian et al. [12]
Prospective, single-center study	2–12	28	2021	Spain	Severe hypoalbuminemia, markedly elevated CRP were associated with inadequate VRC C_trough_.	Valle-T-Figueras et al. [27]
Retrospective, single-center study	< 18	104	2021	China	CRP levels significantly associated with VRC PK in children aged 11–18 years but not in 2–10 years.	Luo et al. [28]
Retrospective, single-center study	< 18	91	2022	China	CYP2C19 phenotypes, CRP concentrations, age, and the presence of immunosuppressants were associated with the VRC PK.	Chen et al. [13]
Retrospective, single-center study	1 to 18	59	2022	China	CYP2C19 phenotypes affected initial VRC C_trough_.	Chen et al. [29]
Retrospective, single-center study	0.5 months to 17	36	2022	Switzerland	CYP2C19 and CYP3A4 polymorphisms and drug transporters ABCC2 and ABCG2, combination medication levetiracetam, ciprofloxacin, and propranolol affected VRC C_trough_.	Tilen et al. [30]
Prospectively single-center study	2 to 14	68	2022	China	VRC C_trough_ of patients with CYP2C19*2 or CYP2C19*3 were significantly higher than that with wild-type carriers.	Fan et al. [31]
Retrospective, single-center study	2 to 14	131	2023	China	CYP2C19 polymorphisms, co-administration of omeprazole, ALB and ALT levels affected VRC C_trough_.	Hu et al. [14]

VRC, voriconazole. C_trough_, trough concentration. ALT, alanine transaminase. ALB, albumin. CRP, c-reactive protein. Scr, serum creatinine. DBil, direct bilirubin. BUN, blood urea nitrogen. WT, weight. PPIs, proton pump inhibitors. Pharmacokinetic, PK.

Genetic factors significantly influenced the metabolism of VRC. Numerous studies have shown a significant correlation between CYP2C19 genetic polymorphism and VRC C_trough_ in pediatric patients. Studies by Espinoza et al. [24] and Fan et al. [31] have found that mutations such as CYP2C19*2 and CYP2C19*3 might lead to decreased enzyme activity and increased VRC C_trough_, while the CYP2C19*17 mutation might result in enhanced enzyme activity and decreased VRC C_trough_. Chen et al.‘s study [29] found that 15.3% of patients were CYP2C19 poor metabolizers (PMs), a proportion higher than that reported in European and American populations. Allegra et al. [23] and Tilen et al. [30] reported that apart from CYP2C19 genotypes, genetic polymorphisms in CYP3A4, SLCO1B3, as well as ABCC2 and ABCG2 also significantly influenced VRC C_trough_.

A retrospective single-center study conducted in 2017 involving Chinese pediatric patients aged 0–12 years demonstrated that concurrent administration of omeprazole significantly elevated VRC C_trough_ (*P* = 0.032), providing the evidence of the impact of omeprazole on VRC C_trough_ in pediatric patients [10]. Hu et al. [11] found that concomitant use of PPIs significantly elevated C_trough_ of VRC (median VRC C_trough_ in patients with and without PPIs co-administration were 2.07 mg/L vs. 0.84 mg/L, respectively, *P* = 0.028) through a retrospective analysis. Co-administration of VRC and PPIs lead to a significant increase in VRC C_trough_.

Currently, four studies have reported the correlation between C-reactive protein (CRP) concentrations and VRC C_trough_ in pediatric patients. A clinical study found differences in the correlation between the CRP concentrations and VRC C_trough_ among pediatric patients of different age groups. Luo et al. [28] discovered a significant correlation between CRP concentrations and VRC PK in pediatric patients aged 11–18 years, but no significant correlation was observed in patients aged 2–10 years.

Due to the nonlinear PK of VRC, C_trough_ could not be predicted by dose. Moreover, most studies indicated that VRC dosage was unrelated to C_trough_. However, the CL of VRC may exhibit linearity in the pediatric population. Liu et al. [10] discovered that no correlation between VRC C_trough_ and dose in pediatric patients aged 2–12 years (*n* = 27, *r* = 0.151, *P* = 0.452), however, a notable correlation was observed between VRC C_trough_ and dosage (*n* = 74, *r* = 0.370, *P* = 0.001) in pediatric patients < 2 years old. Allegra et al. [22] also found a significant correlation between VRC C_trough_ and dosage in pediatric patients (*n* = 237, *r* = 0.195, *P* = 0.016) in pediatric patients < 18 years old. Hence, the PK of VRC in pediatric patients may differ from those in adults.

VRC C_trough_ were related to the routes of administration. Several studies have explored the impact of administration routes on VRC C_trough_. Patients receiving intravenous administration exhibited significantly higher VRC C_trough_ compared to those receiving oral administration. Research by Allegra et al. demonstrated a positive correlation between VRC C_trough_ and age in 237 Italian pediatric patients [22]. Furthermore, VRC C_trough_ may also be associated with gender, liver and kidney function indicators. Allegra et al. also found that VRC C_trough_ were significantly higher in males compared to females [22], while Liu et al. confirmed a significant positive correlation between VRC C_trough_ and serum creatinine (Scr), and a significant negative correlation with serum albumin (ALB) levels [10]. Kang et al. [25] found a significant positive correlation between VRC C_trough_ and aspartate aminotransferase (AST) levels.

### What recommendations can be derived from the PPK study of VRC for personalized medication?

Currently, a total of 11 studies have established PPK models for pediatric patients. Nine studies used a two-compartment model and the most commonly used tool in PPK studies was non-linear mixed effect modeling (NONMEM). Among the 11 studies, 8 studies utilized NONMEM and only one PPK model incorporated CRP concentrations into covariance analysis [41], as detailed in Table 4. High inter-individual variability in VRC PK among the pediatric population had been revealed. Most of studies have identified CYP2C19 genetic polymorphisms as significant covariates influencing the PK parameters of VRC [32, 33, 35, 39, 40, 42]. Furthermore, covariates including body weight, age, CRP concentrations, co-administration of omeprazole, and liver function indicators such as ALB, alanine transaminase (ALT), and alkaline phosphatase (ALP) levels, may also be associated with VRC PK parameters [32–36, 39–42].

**Table 4:** Summary of studies on PPK analysis and current recommendations of VRC optimal dosing regimen in pediatric patients

Study design	No. of samples	Age (years)	Reference	Country of study populations	Year	Target C_trough_ (mg/L)	Software	Modeling	Significant covariates	The results of dose simulation or the recommendations of optimal dose regimen
An open, multicenter, two-cohort study	35	2–11	Walsh et al. [32]	America, Costa Rica, Panama, and Britain	2004	/	NONMEM	A two-compartment disposition	WT, CYP2C19 genotype, ALT and ALP	4 mg/kg required in children to achieve exposures was consistent with those in adults following 3 mg/kg
Data from three open-label studies	82	2-<12	Karlsson et al. [33]	/	2009	/	NONMEM	A two-compartment disposition	CYP2C19 genotype and ALT	7 mg/kg IV or 200 mg PO q12h
A prospective study	46	8 months-20.5	Neely et al. [34]	America	2010	/	MM-USCPACK	A two-compartment Michaelis-Menten	Age	7 mg/kg IV or 200 mg PO q12h
Data from 5 previous PK studies	112	2-<12	Friberg et al. [35]	/	2012	/	NONMEM	A two-compartment with first-order absorption and mixed linear and nonlinear elimination	WT, age and CYP2C19 genotype	The IV loading dose of 9 mg/kg in children to attain exposures was comparable to that in adults receiving 6 mg/kg IV. Dosages of 4 and 8 mg/kg IV q12h in children were akin to those in adults receiving 3 and 4 mg/kg IV q12h. The 9 mg/kg PO (maximum, 350 mg) q12h paralleled the adult regimen of 200 mg PO q12h.
An open-label, multicenter, phase II study	21	3–14	Muto et al. [36]	Japan	2015	/	NONMEM	A two-compartment with first-order absorption and mixed linear and nonlinear elimination	Age and WT	/
A phase II study	23	0.5–21	Gastine et al. [37]	Germany	2018	1.0–6.0	NONMEM	A two-compartment with first-order absorption, nonlinear Michaelis-Menten elimination	/	9 mg/kg IV TID for up to 3 days
A retrospective study	55	≤ 18	Carlesse et al. [38]	Brazil	2019	1.0–6.0	Pmetrics	A nonparametric population	/	/
A single-institution, phase I study	58	≤ 21	Takahashi et al. [39]	America	2021	1.5-5.0	NONMEM	A two-compartment parent mixed linear/nonlinear	WT and CYP2C19 phenotype	For NMs: 16 mg/kg (< 15 kg), 12 mg/kg (15–30 kg), or 10 mg/kg (> 30 kg). Doses for PMs were 33–50% lower, while for UMs, doses were 25–50% higher.
A retrospective study	99	0.44–13.58	Wang et al. [40]	China	2021	1.0-5.5	Phoenix NLME	A two-compartment with nonlinear Michaelis-Menten elimination	WT, CYP2C19 phenotype and omeprazole	For most children, two loading doses of 9 mg/kg q12h were recommended, while for children weighing ≤ 18 kg, three loading doses of 6-7.5 mg/kg q8h were suggested (except for PMs). The maintenance doses in PMs were reduced by about 30–40% compared to NMs.
A single-institution, phase I study	59	< 21	Takahashi et al. [41]	America	2022	/	NONMEM	A two-compartment linear elimination	CRP and ALB	/
A retrospective study	67	1.08–17.92	Wu et al. [42]	China	2022	0.5-5.0	NONMEM	A one-compartment with first-order absorption and elimination	WT, CYP2C19 phenotype and ALB	Order of the recommended doses: NM > IM > PM. Children with lower WT should receive a higher dose, while those with lower ALB levels should receive a lower dose.

VRC, voriconazole. PPK, population pharmacokinetics. PK, pharmacokinetic. C_trough_, trough concentration. PO, oral. IV, intravenous. UMs, ultrarapid metabolizers. NMs, normal metabolizers. IMs, intermediate metabolizers. PMs, poor metabolizers. WT, weight. ALT, alanine transaminase. ALP, alkaline phosphatase. ALB, albumin. CRP, C-reactive protein. BID, twice times a day. TID, three times a day

Some studies employed the final models to explore optimal dosing regimens through dose simulation experiments. For instance, studies by Takahashi et al. [39], Wang et al. [40], and Wu et al. [42] proposed dose recommendations based on body weight and CYP2C19 genetic polymorphisms. All three studies recommended lower VRC doses for CYP2C19 PMs. Karlsson et al. [33] and Gastine et al. [37] directly provided simple and unified dose recommendations. Studies by Walsh et al. [32] and Friberg et al. [35] suggested dosing regimens in pediatric patients to achieve VRC exposures comparable to those in adults. Moreover, some studies proposed dose optimization suggestions based on other significant covariates. Wang et al. [40] suggested a slight reduction in VRC dose when co-administered with omeprazole, while Wu et al. [42] proposed that children with lower body weight might require higher VRC doses and those with low ALB levels might need lower VRC doses. By comparing estimated PK parameters between adults and pediatric patients, we found that PK parameters in children might differ from those in adults. Muto et al.‘s study [36] investigated the metabolic characteristics of VRC in Japanese pediatric immunocompromised patients, revealing an average bioavailability of 73% in this group, whereas it was 96% in healthy adult patients. Gastine et al.‘s study [37] estimated an average bioavailability of 59.4%. However, Wu et al.‘s study [42], which focused on the Chinese pediatric population, demonstrated that the bioavailability in pediatric patients could reach 90.2%.

## Discussion

At present, research concerning the utilization of VRC in pediatric patients is garnering heightened attention. Investigations into the factors influencing VRC C_trough_, along with PPK analyses, serve as pivotal guides for dose optimization. Nonetheless, the realm of VRC utilization in pediatric patients with challenges like limited sample sizes and a preponderance of retrospective studies. These hurdles underscore the necessity for further comprehensive exploration within this special population.

### i. What dosage is required to attain the target C_trough_ of VRC?

Differences in VRC dosing exist between Asian and non-Asian pediatric patients, which may be attributed to variations in genetic backgrounds between these populations. Since VRC was predominantly metabolized by the liver enzyme CYP2C19, the proportion of Asians with the CYP2C19 PMs ranged from 15 to 20%, whereas in Caucasians, it was 3–5% [43]. This divergence could lead to differences in VRC metabolism among different ethnicities and subsequently resulted in variations in the required dosages to achieve target C_trough_. Asian pediatric patients may not be suited for the recommended dosages stated in the original manufacturer’s instructions.

The latest consensus by the JSC/JSTDM (2022) [44] suggested the necessity of reducing the standard dose for Asian populations due to the observed high incidence of supertherapeutic concentrations in TDM practice in Japan. Moreover, the consensus emphasized the need for distinct dosing regimens tailored to Asian and non-Asian populations to prevent overdosing. In the future, it is hoped that large-scale, cross-ethnicity prospective studies will be conducted to explore optimal dosages of VRC for diverse pediatric populations worldwide.

In addition, studies have indicated that pediatric patients needed to be administered appropriate dosages based on their age. Younger children may exhibit higher CL of VRC compared to older children, potentially necessitating different VRC doses among age groups. Nevertheless, guidelines have yet to specify reference VRC doses for pediatric patients (< 6, 6–12, > 12 years old). Furthermore, according to the FDA drug label information [45], it was important to consider that pediatric patients may have shorter gastrointestinal transit times, possibly affecting tablet absorption compared to adults. As a result, oral suspension was recommended for pediatric patients aged 2 to 12 years. However, the bioequivalence or PK studies between oral tablets and suspension of VRC has not been investigated in pediatric populations.

### ii. What factors influence VRC C_trough_ in pediatric patients?

When assessing factors influencing VRC C_trough_, although most of the studies were retrospective and single-center, they confirmed the already well-known factors such as CYP2C19 polymorphisms, concurrent use of PPIs, and patient age. Additionally, new factors including other genetic polymorphisms, CRP concentration, liver and kidney function, as well as gender, have been identified.

Weiss et al. proposed that CYP2C19 genotype significantly contributed to the high variability observed in VRC PK [46]. Trubiano et al. [47] also suggested that the CYP2C19 genotype could be utilized to predict VRC C_trough_ and toxicity. Many studies suggested using CYP2C19 genotype to guide the initial dosing regimen of VRC [48, 49]. A study involving prophylactic use of VRC in acute myeloid leukemia patients found that CYP2C19 genotype testing not only avoided prolonging hospital stays but also moderately reduced costs, and it was projected that each patient could save $ 415 in hospitalization expenses [49].

The variability of VRC C_trough_ can not be fully explained by concomitant medications, genetic polymorphisms of metabolic enzyme, or liver disorders. Recent researches indicated a correlation between elevated CRP concentration and lower VRC C_trough_. Morgan et al. [50] suggested that the release of cytokines upon inflammatory stimulation altered the activity of transcription factors in the liver. These alterations lead to the downregulation of most CYP genes, affecting the production of metabolic proteins and subsequently reducing the CL of VRC. In vitro studies have provided compelling evidence indicating that pro-inflammatory cytokines, especially interleukin-1 (IL-1), IL-6, and tumor necrosis factor-alpha (TNF-α), downregulated the biosynthesis of CYP isoforms, including CYP2C19, CYP3A4, and CYP2C9, which play pivotal roles in VRC metabolism [51, 52]. The correlation between CRP concentrations and VRC C_trough_ showed variations in different age groups of pediatric patients. This discrepancy may be attributed to the distinct roles of CYP2C19, CYP3A4, and flavin-containing monooxygenase 3 (FMO-3) in VRC N-oxidation between pediatric patients and adults. Studies have found that the CL of VRC in patients aged 2 to 11 years was nearly three times that of adults [47]. CYP2C19 and FMO-3 exhibited higher metabolic activity in young children, and the downregulation of CYP2C19 isoforms during inflammation had a relatively minor impact on VRC metabolism in younger children. Further research is needed to explore how to achieve personalized dosing of VRC based on inflammatory status.

Although CYP2C19 enzymes accounted for only 5% of drug metabolism [46], they were involved in the metabolism of various drugs such as PPIs, antiepileptic drugs, antiplatelet drugs, and antidepressants. PPIs and corticosteroids being the most studied drugs that interact with VRC. The guideline issued by the Chinese Pharmacological Society (CPS) recommended closely monitoring the efficacy and safety of VRC when administered concomitantly with PPIs or corticosteroids [53].

The VRC C_trough_ in pediatric patients were correlated with indicators of hepatic and renal function, indicating that elevated VRC C_trough_ might be linked to impaired hepatic and renal function. For pediatric patients with normal renal function, the drug label recommended intravenous treatment for at least the initial 7 days of therapy for those with IA. Subsequently, upon clinical improvement and tolerance of oral medication, the oral tablet or suspension forms of VRC may be utilized. However, injectable VRC with the solvent sulfobutylether-β-cyclodextrin has been associated with adverse effects on kidney function due to potential accumulation. Research conducted by Yasu et al. [54] has demonstrated a significant correlation between renal function deterioration and cumulative intravenous VRC dose (≥ 400 mg/kg). These findings indicated that higher cumulative intravenous VRC doses may contribute to the risk of impaired kidney function. The FDA drug instructions advised careful attention was required when administering VRC intravenous preparations to patients with renal insufficiency (creatinine clearance rate < 50 ml/min) [45]. However, the long-term effects of intravenous VRC use on kidney function remain unclear. Currently, there is limited research on the use of VRC in pediatric patients with impaired hepatic or renal function.

### iii. What recommendations can be derived from the PPK study or guidelines of VRC for personalized medication?

Despite the high inter-individual PK variability of VRC, PPK software for individualized dosing can accurately simulate VRC C_trough_, with predicted levels closely aligning with actual measured values. PPK model may be an immensely useful tool for further optimizing VRC dosing and assisting in TDM for clinical therapies. Further prospective research is required to determine its role in clinical practice. Utilizing the PPK model to describe patients’ PK characteristics and examining covariates significantly influencing VRC C_trough_ can provide essential information for formulating individualized dosing regimens. The guideline of CPS recommended adjusting VRC dosing based on a PPK model for the Chinese population [53]. Therefore, PPK analysis for VRC in children is an important direction in future research.

Numerous PPK studies have emphasized CYP2C19 polymorphism as a significant covariate influencing the PK parameters of VRC, and some have proposed dosing regimens based on different CYP2C19 genotypes through dose simulation experiments. However, determining the initial dose by detecting CYP2C19 genotype is not yet recommended in the FDA drug label. The Clinical Pharmacogenetics Implementation Consortium (CPIC) guideline [55] provided dosing optimization schemes for VRC treatment based on CYP2C19 phenotype in patients aged < 18 years. For CYP2C19 rapid metabolizers (RMs), normal metabolizers (NMs), and intermediate metabolizers (IMs), initiating treatment with standard doses was recommended, with TDM advised for RMs to adjust the dose to achieve therapeutic C_trough_. In cases where VRC use was unavoidable for PMs, a reduced standard dose and TDM were recommended. Ultra-rapid metabolizers (UMs) were advised to switch to alternative drugs that did not undergo CYP2C19 metabolism, such as amphotericin B and posaconazole.

Previous research had proposed that the CL of VRC in pediatric patients was three times that of adults [56]. Studies by Pascual et al. [57] and Wang et al. [58] on adult patients reported VRC CL of 5.2 L/h and 6.95 L/h, respectively. However, research by Takahashi et al. estimated a VRC CL of 12.3 L/h for pediatric patients. Numerous PPK studies also suggested that pediatric patients often require higher doses than adults in order to achieve the same VRC exposure. In the 2013 Guideline for Japan [59], children were recommended to receive a dosage of 7 mg/kg q12h, which was lower than the dosage specified in the FDA drug label information. However, both the ESCMID-ECMM [60] and UK [61] guidelines advocated a loading dose of 9 mg/kg q12h, followed by a maintenance dose of 8 mg/kg q12h for the intravenous preparation, with oral dosing maintained at 9 mg/kg q12h, consistent with the dosage stated in the original manufacturer’s instructions. The latest consensus suggested that altering the initial VRC dose when coadministered with PPIs might be unnecessary until the results of TDM were available. The impact of CRP levels on the VRC C_trough_ has been confirmed in numerous studies. However, many PPK studies of VRC did not include CRP concentrations. Hence, future PPK studies should consider incorporating inflammatory indicators such as CRP concentrations.

## Conclusions

In recent years, due to the widespread of TDM and CYP2C19 genotype testing for VRC, the realization of VRC personalized therapies has become a prominent research focus. VRC C_trough_ exhibit high inter- and intra-individual variability, potentially influenced by various factors such as age, concomitant medications, inflammatory status, hepatic and renal functions, as well as genetic polymorphisms in metabolic enzyme. Some unknown influencing factors need to be explored in the further studies. It is anticipated that more studies on personalized therapy of VRC will emerge, contributing to a comprehensive understanding of the factors influencing VRC C_trough_ and PK variability.

## Data Availability

Not applicable.
